# Possibility of adiponectin use to improve islet transplantation outcomes

**DOI:** 10.1038/s41598-021-04245-0

**Published:** 2022-01-10

**Authors:** Naoaki Sakata, Gumpei Yoshimatsu, Kiyoshi Chinen, Ryo Kawakami, Shohta Kodama

**Affiliations:** 1 Department of Regenerative Medicine and Transplantation, Faculty of Medicine, Fukuoka University, 7-45-1 Nanakuma, Jonan-ku, Fukuoka, Fukuoka 814-0180 Japan; 2 Center for Regenerative Medicine, Fukuoka University Hospital, 7-45-1 Nanakuma, Jonan-ku, Fukuoka, Fukuoka 814-0180 Japan

**Keywords:** Translational research, Cell transplantation, Diabetes, Pancreas

## Abstract

Although islet transplantation (ITx) is a promising therapy for severe diabetes mellitus, further advancements are necessary. Adiponectin, an adipokine that regulates lipid and glucose metabolism, exerts favorable effects on islets, such as reinforcement of the insulin-releasing function. This study evaluated the possibility of adiponectin use to improve ITx outcomes. We treated mouse islets with 10 µg/mL recombinant mouse adiponectin by overnight culture and then assessed the insulin-releasing, angiogenic, and adhesion functions of the islets. Furthermore, 80 syngeneic islet equivalents with or without adiponectin treatment were transplanted into the renal subcapsular space of diabetic mice. In in vitro assessment, released insulin at high glucose stimulation, insulin content, and expressions of vascular endothelial growth factor and integrin β1 were improved in adiponectin-treated islets. Furthermore, adiponectin treatment improved the therapeutic effect of ITx on blood glucose levels and promoted angiogenesis of the transplanted islets. However, the therapeutic effect was not pronounced in glucose tolerance test results. In conclusion, adiponectin treatment had preferable effects in the insulin-releasing, angiogenic, and adhesion functions of islets and contributed to the improvement of ITx. The future use of adiponectin treatment in clinical settings to improve ITx outcomes should be investigated.

## Introduction

Islet transplantation (ITx) is a promising therapy for patients with severe diabetes mellitus (DM) suffering from severe hypoglycemic events. Generally, clinical ITx is performed by intraportal islet infusions for engraftment in the liver. A recent multicenter clinical trial for patients with type 1 DM with normal renal function complicated by severe hypoglycemia (Clinical Islet Transplantation 07) revealed that approximately 90% of the participants showed improved levels of hemoglobin A1c and hypoglycemic events were prevented^1^. However, the efficacy of ITx is inferior to that of pancreatic organ transplantation. In most cases, two or three ITxs are recommended to stabilize blood glucose levels and allow the withdrawal of insulin treatment^2^. The inferiority of ITx is mainly due to an instant blood-mediated inflammatory reaction with acute thrombosis that is induced by activation of complement, coagulation, and innate immunity, which impairs transplanted islets^3^. Furthermore, there is ischemia of the islet engraftment until the vascular network formation is complete^4^. These factors lead to the deterioration of transplant efficacy^5,6^. Therefore, there is a need for novel supportive treatments that promote islet engraftment.

Adiponectin is a major adipokine that circulates in the blood vessels^7^ and is produced by both white and brown adipose tissues. Adiponectin has several metabolic functions in lipid synthesis and storage, neoglucogenesis, and peripheral glucose utilization. It plays a vital role in preventing insulin resistance in skeletal muscle by improving its insulin-sensitizing properties^7^, and its antiatherogenic function prevents dyslipidemia^8^. Yamauchi et al. revealed that adiponectin administration ameliorated hyperglycemia in lipoatrophic mice by compromising insulin resistance in skeletal muscle and liver^9^. They also clarified that adiponectin functions occurred via adiponectin receptors (AdipoR1 and AdipoR2)^10^, which are expressed not only in skeletal muscle or liver^10,11^ but also in islets^11^. Recent studies have elucidated the antiapoptotic effect of adiponectin on islets^12^ and its insulin-releasing function^13^ via the adiponectin receptors, which may improve ITx outcomes. However, the potential of adiponectin for this treatment has not been investigated in detail. Therefore, this study evaluated the possibility of adiponectin use to improve ITx outcomes.

## Results

### Adiponectin receptors were strongly expressed on isolated islets

**Figure 1:**
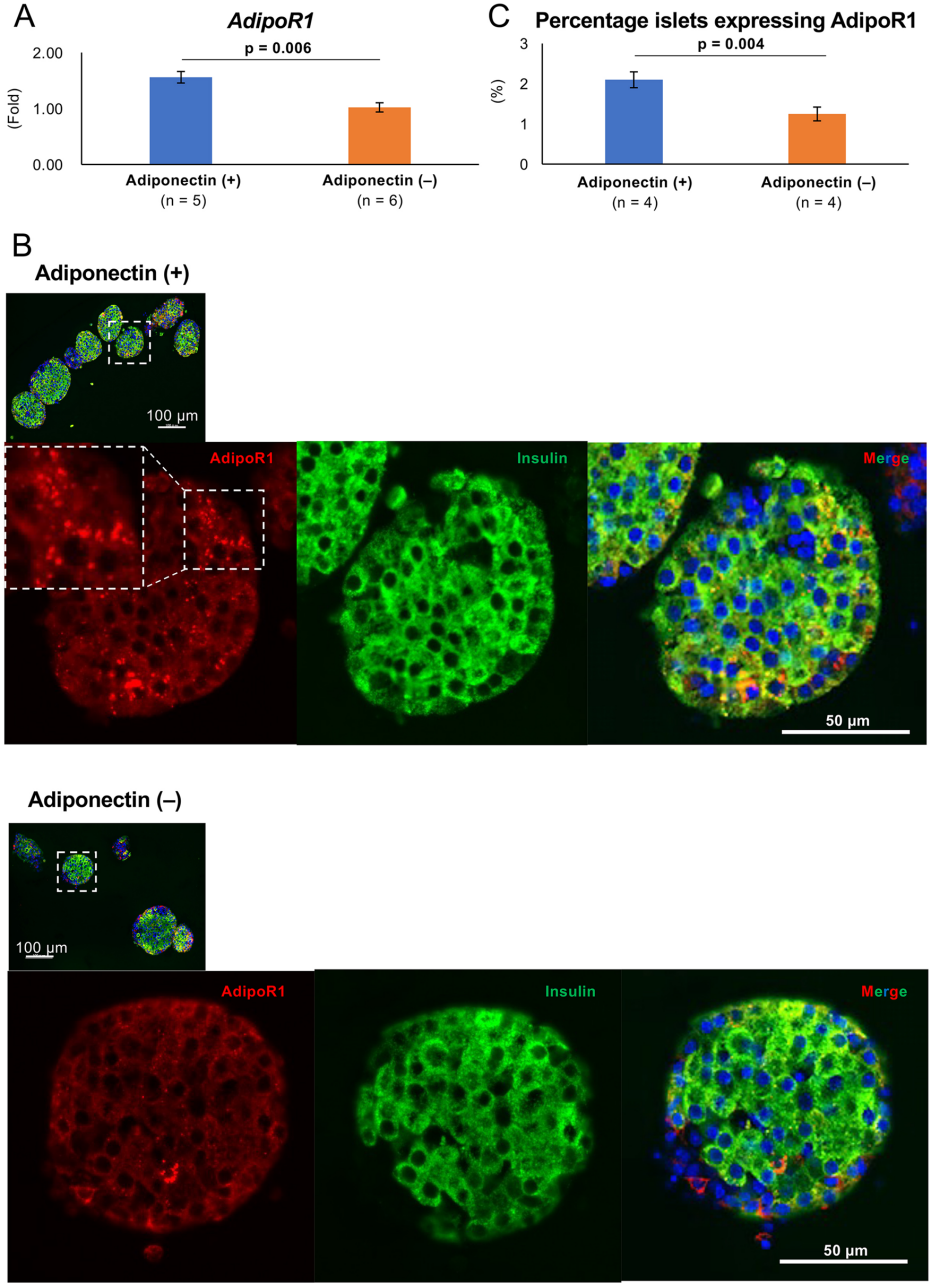
AdipoR1 expression on isolated islets. (**A**) *AdipoR1* expression in islets with adiponectin treatment (adiponectin (+), blue) and without adiponectin treatment (adiponectin (−), orange) was quantified by qRT-PCR. The results were normalized to the β-actin housekeeping gene and were presented as the fold difference over the detectable Ct value, which was calculated using the ΔΔCt method. (**B**) Immunohistochemical findings of isolated islets in the adiponectin (+) group (upper) and adiponectin (−) group (lower) stained for AdipoR1 (red), insulin (green), and nuclei (blue, using DAPI). The AdipoR1 regions expressing β cells (yellow) are indicated by white triangles. Scale bar: 100 µm and 50 µm. (**C**) Quantification of AdipoR1-positive area per islet (percentage islets expressing AdipoR1). Data are presented as means ± standard error of the mean. A *p*-value < 0.05 was considered statistically significant.

At the beginning of this study, we confirmed the expression of adiponectin receptors in isolated mouse islets with or without adiponectin treatment. At the gene level, the expression of both *AdipoR1* and *AdipoR2* were detected in islets in the adiponectin (+) (islets with adiponectin treatment) and adiponectin (−) (islets without adiponectin treatment) groups (Fig. 1A, Supplemental Fig. 1A). The *AdipoR1* expression was significantly higher in the adiponectin (+) group (1.55 ± 0.10 vs. 1.02 ± 0.08, *p* = 0.006; Fig. 1A). Immunohistochemistry staining for AdipoR1 and AdipoR2 was performed on the isolated islets to visualize the islet receptors. Figure 1B shows the immunohistochemical results of islets with or without adiponectin treatment. AdipoR1 receptors were observed on the outer membrane of endocrine cells, including β cells (detected as yellow regions) in islets with and without adiponectin treatment. The AdipoR1-positive area appeared wider in the islets with adiponectin treatment and was more evident using image analysis. The percentage of AdipoR1-positive areas per islet (percentage islets expressing AdipoR1) was significantly higher in the adiponectin (+) group (2.10 ± 0.20% vs. 1.24 ± 0.17%, *p* = 0.004; Fig. 1C). Moreover, AdipoR2 expression was weaker than that of AdipoR1, and no difference was noted in the percentage of AdipoR2-positive areas (percentage islets expressing AdipoR2) between the two groups (0.89 ± 0.13% vs. 0.76 ± 0.13%; Supplemental Fig. 1B,C).

### Adiponectin treatment improved the insulin-releasing function of islets

**Figure 2:**
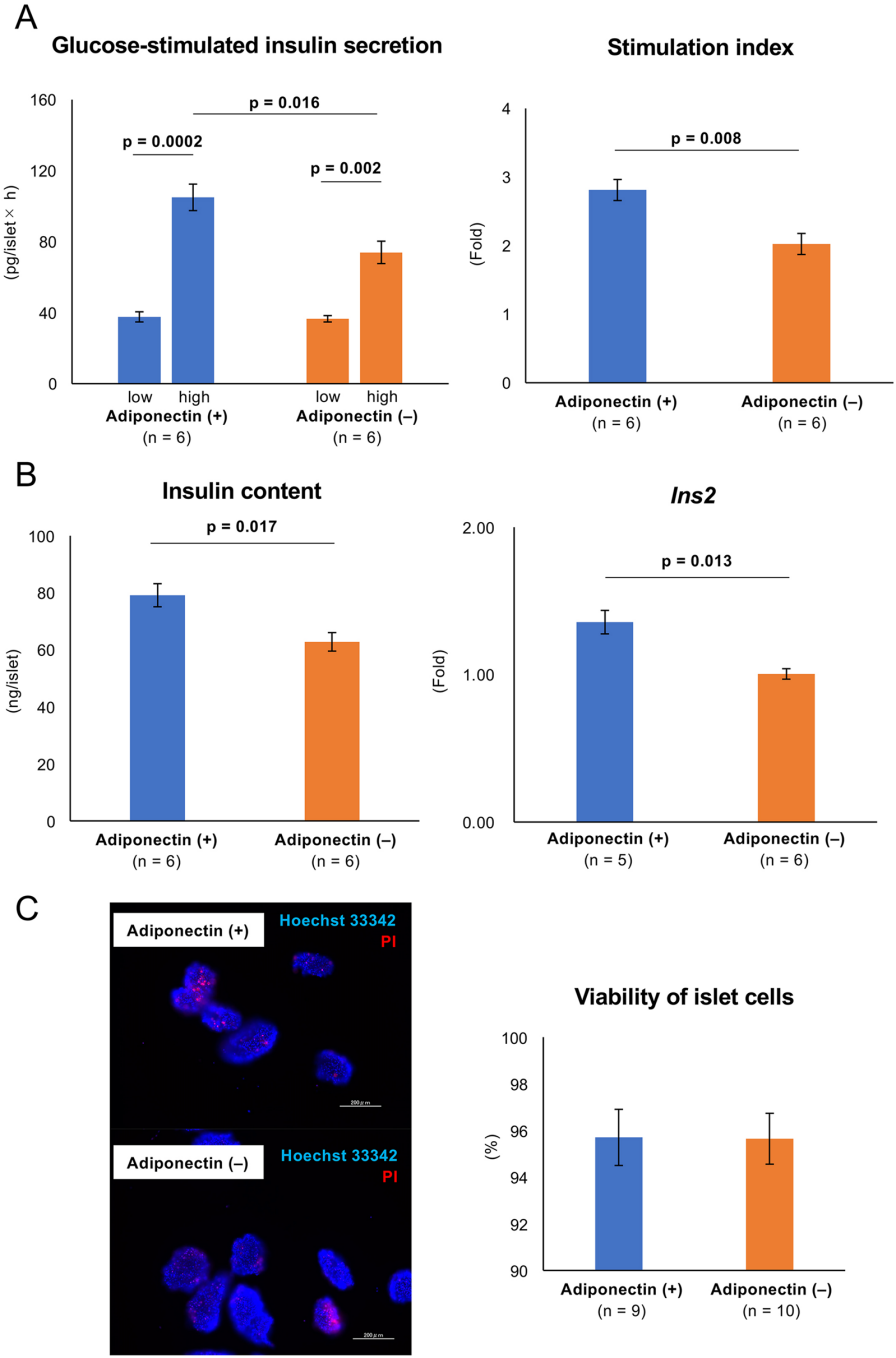
GSIS, volume of insulin in islets, and viability of islets. GSIS (**A**), volume of insulin (**B**), and viability (**C**) of islets treated with adiponectin (adiponectin (+), blue) and without adiponectin (adiponectin (−), orange). (**A**) Amount of insulin released from islets under conditions of low and high glucose stimulation (left) and the stimulation index (right). (**B**) Insulin content in islets (left) and *Ins2* expression in islets quantified by qRT-PCR (right). The results were normalized to the β-actin gene and are presented as the fold difference over the detectable Ct value, which was calculated using the ΔΔCt method. (**C**) Staining to evaluate the viability of islet cells (left). Hoechst 33342 (blue) was used to stain all islet cells, and propidium iodide (PI; red) stained all dead islet cells. Cellular viability of islets (right). Data are presented as means ± standard error of the mean. A *p*-value < 0.05 was considered statistically significant.

Next, we evaluated whether adiponectin treatment promoted the insulin-releasing function of islets. The glucose-stimulated insulin secretion (GSIS) data revealed that the level of insulin released under conditions of high glucose stimulation was significantly higher in islets in the adiponectin (+) group (104.97 ± 7.45 vs. 73.96 ± 6.31 pg/islet × h, *p* = 0.016; Fig. 2A). There was no difference in the amount of insulin released under conditions of low glucose stimulation between the two groups (37.63 ± 2.86 vs. 36.55 ± 1.80 pg/islet × h; Fig. 2A). The stimulation index was significantly higher in islets in the adiponectin (+) group (2.81 ± 0.15 vs. 2.02 ± 0.15, *p* = 0.008; Fig. 2A). Furthermore, islets in the adiponectin (+) group contained a higher insulin content (79.12 ± 4.05 vs. 62.75 ± 3.24 ng/islet, *p* = 0.017; Fig. 2B). Regarding *Ins2* (gene codes for insulin) expression, the level was also significantly higher in islets in the adiponectin (+) group (1.36 ± 0.08 vs. 1.00 ± 0.04, *p* = 0.013; Fig. 2B). These data indicated that adiponectin improved the islets’ insulin-releasing function (depending on glucose concentration) and might promote insulin production. Furthermore, the viability of islets treated overnight with adiponectin was similar to that of islets without adiponectin treatment (high levels of viability), indicating that adiponectin did not impair viability (95.70 ± 1.20% vs. 95.65 ± 1.09%; Fig. 2C).

### Adiponectin may promote angiogenesis of transplanted islets

**Figure 3:**
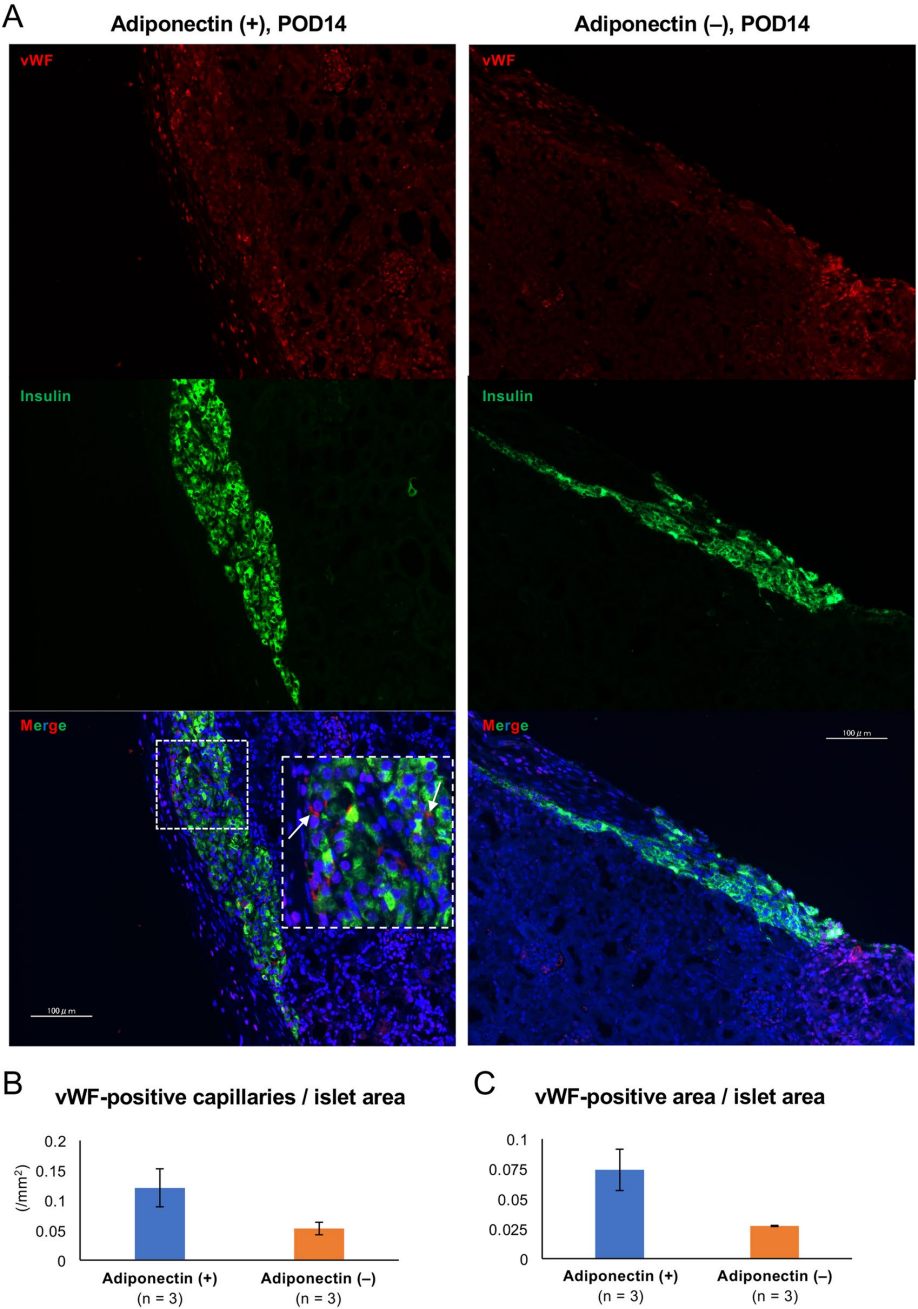
Angiogenesis in transplanted islets. (**A**) Immunohistochemical findings of transplanted islets in the renal subcapsular space at POD 14. Islets treated with adiponectin (adiponectin (+), left) and without adiponectin (adiponectin (−), right) were stained for insulin (green) and vWF (red, indicated by arrows). DAPI was used for nuclear staining (blue). (**B**) Vessel densities, defined as the number of vWF-positive capillaries per islet area, at POD 14. (**C**) Vessel densities, defined as the vWF-positive area per islet area, at POD 14. Data are presented as means ± standard error of the mean. A *p*-value < 0.05 was considered statistically significant.

**Figure 4:**
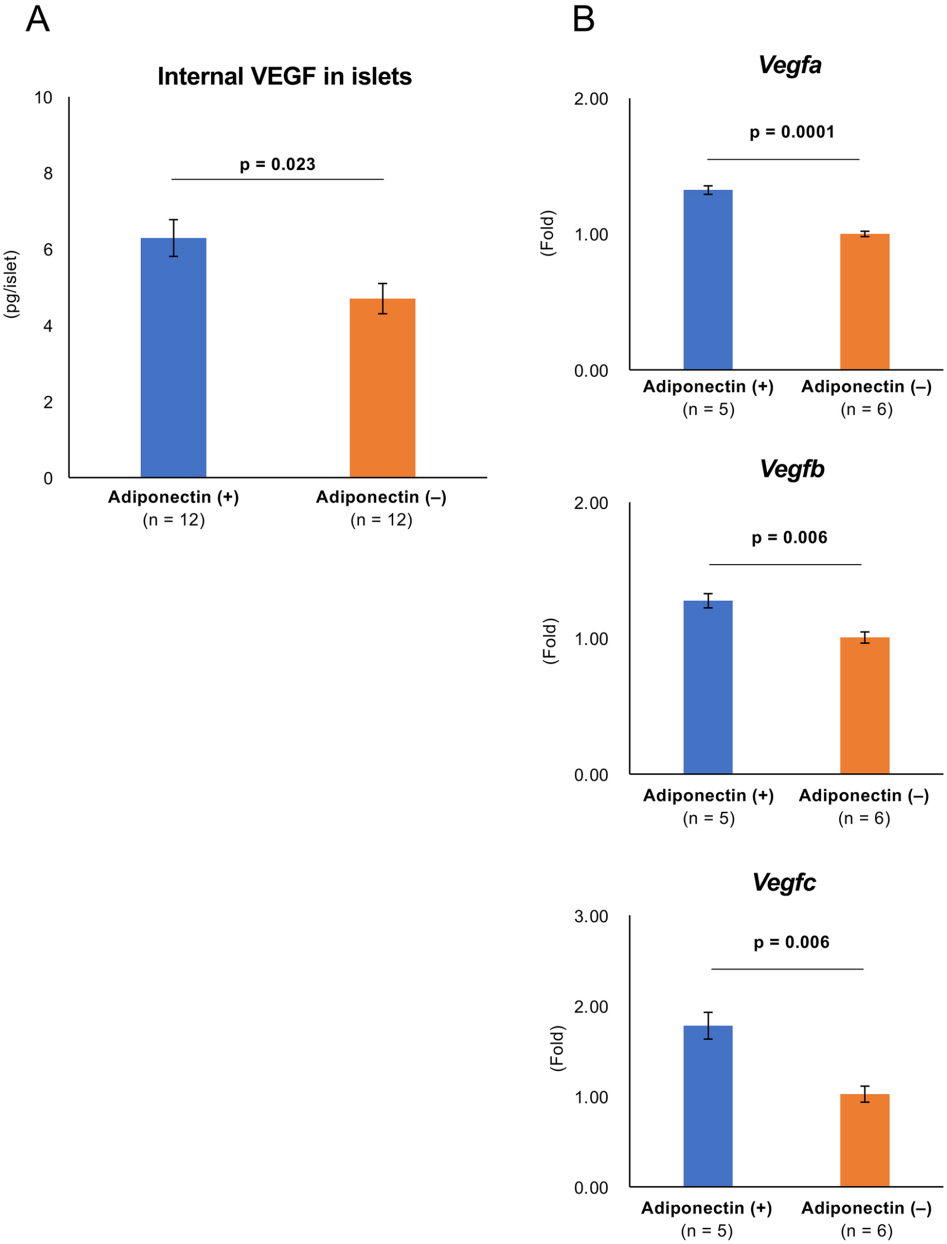
Expressions of angiogenic factors in islets. (**A**) Internal VEGF in islets treated with adiponectin (adiponectin (+), blue) and without adiponectin (adiponectin (−), orange). (**B**) The expression of *Vegfa* (upper), *Vegfb* (middle), and *Vegfc* (lower) in islets as quantified by qRT-PCR. The results were normalized to the β-actin gene and are presented as the fold difference over the detectable Ct value, which was calculated using the ΔΔCt method. Data are presented as means ± standard error of the mean. A *p*-value < 0.05 was considered statistically significant.

We evaluated the angiogenic effect of adiponectin because new vessel formation increased significantly in transplanted islets in white adipose tissue compared with islets transplanted into the renal subcapsular space in a previous study^14^. Figure 3A shows the immunohistochemical results of transplanted islets in the renal subcapsular space at postoperative day (POD) 14 in the adiponectin (+) and adiponectin (−) groups, where prominent angiogenesis (increased von Willebrand factor [vWF]-positive capillaries and area) was observed in the adiponectin-treated islets (Fig. 3A–C). In contrast, there were no significant differences in both vWF-positive capillaries and area between the two groups at POD 56 (Supplemental Fig. 2). Thus, adiponectin treatment may promote the production of angiogenic factors in islets. Therefore, the expression of angiogenic factors was assessed at the protein and gene levels. The assessment clarified that internal vascular endothelial growth factor (VEGF) in islets was significantly increased by adiponectin treatment (6.29 ± 0.48 vs. 4.70 ± 0.40, *p* = 0.023; Fig. 4A). Regarding gene levels, the higher levels of expression of *Vegfa*, *Vegfb*, and *Vegfc* were observed in the adiponectin (+) group (1.32 ± 0.03 vs. 1.00 ± 0.02, *p* = 0.0001*;* 1.27 ± 0.05 vs. 1.00 ± 0.04, *p* = 0.006; and 1.78 ± 0.15 vs. 1.02 ± 0.09, *p* = 0.006; respectively) (Fig. 4B).

### Adiponectin may promote the expression of adhesion factors in transplanted islets

**Figure 5:**
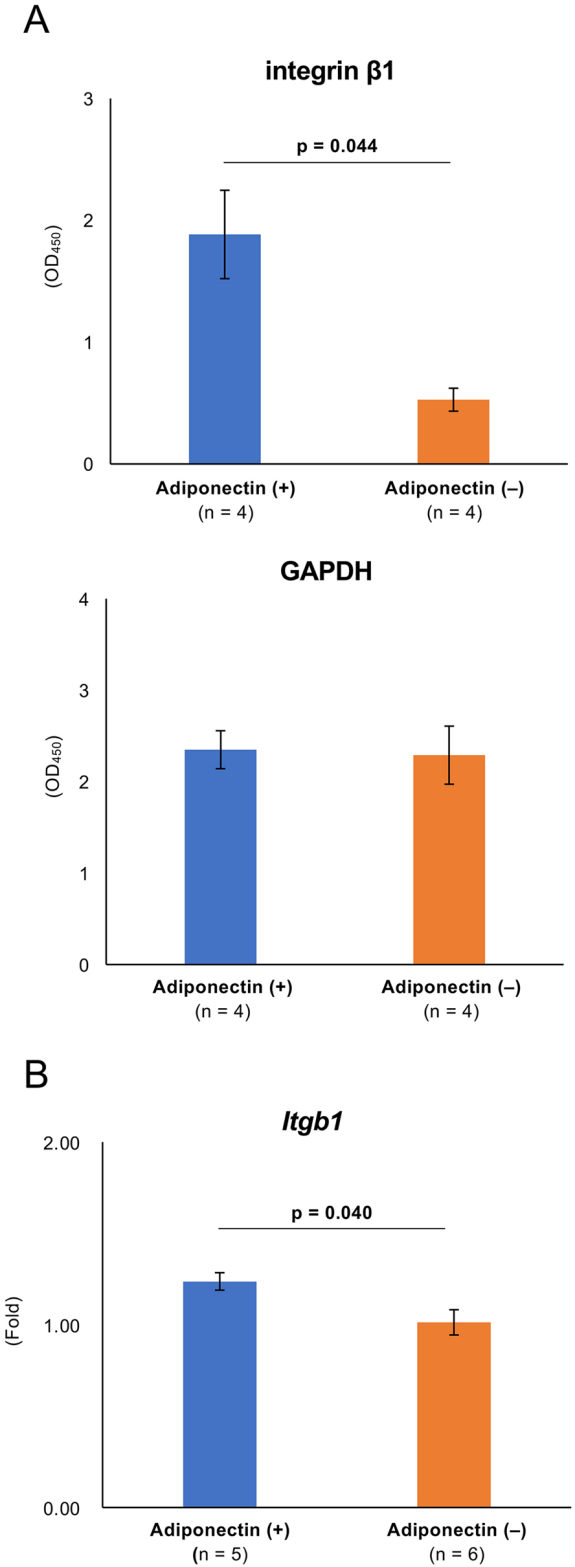
Expression of adhesion factors in islets. (**A**) Expression of integrin β1 (upper) and GAPDH (lower) on islets treated with adiponectin (adiponectin (+), blue) and without adiponectin (adiponectin (−), orange). GAPDH was used as an internal control. (**B**) *Itgb1* expression in islets quantified by qRT-PCR. The results were normalized to the β-actin gene and are presented as the fold difference over the detectable Ct value, which was calculated using the ΔΔCt method. Adiponectin (+) (blue), adiponectin (−) (orange). Data are presented as means ± standard error of the mean. A *p*-value < 0.05 was considered statistically significant.

We then attempted to clarify whether adiponectin promoted the expression of adhesion factors in islets. The level of integrin β1 expression was significantly higher in islets in the adiponectin (+) group compared with those in the adiponectin (−) group (optical density at 450 nm [OD_450_]: 1.89 ± 0.36 vs. 0.53 ± 0.09, *p* = 0.044; Fig. 5A), while there was no difference in the expression of glyceraldehyde 3-phosphate dehydrogenase (GAPDH), which was used as an internal control (Fig. 5A). Regarding gene assessment, the *Itgb1* (gene codes for integrin subunit β1 protein) expression was significantly higher in islets in the adiponectin (+) group than those in the adiponectin (−) group, and similar results were observed at the protein level (1.24 ± 0.05 vs. 1.01 ± 0.07, *p* = 0.040; Fig. 5B).

### Adiponectin partially contributed to an improvement of the therapeutic effect of ITx

**Figure 6:**
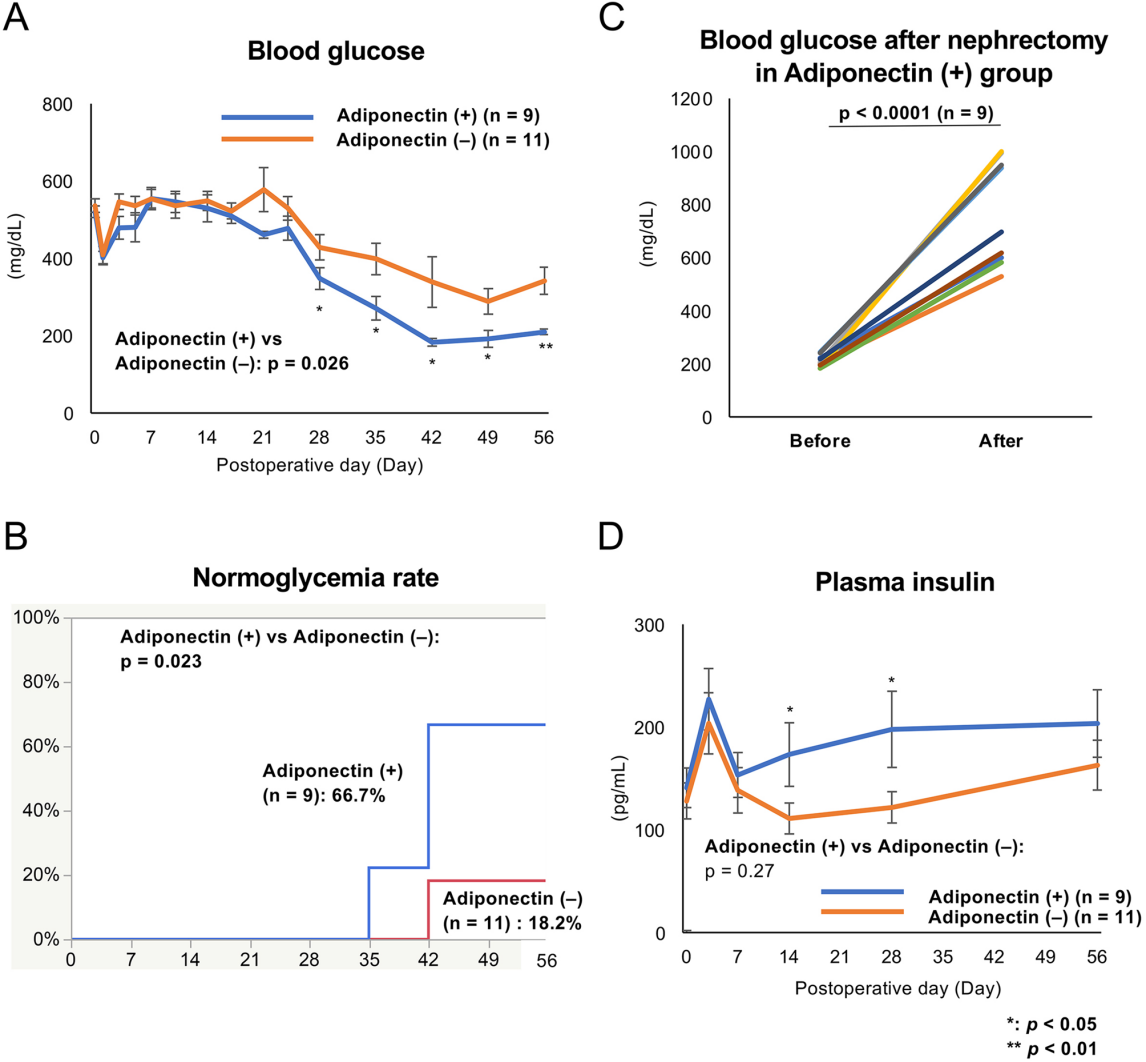
The therapeutic effect of adiponectin treatment in ITx. Blood glucose (**A**) and plasma insulin (**D**) levels after ITx in the islets treated with adiponectin (adiponectin (+), *n* = 9) and without adiponectin (adiponectin (−), *n* = 11). Normoglycemia was defined as a blood glucose level < 200 mg/dL. The rate of normoglycemia is shown in (**B**). The change of blood glucose level in the adiponectin (+) group before and after nephrectomy was also assessed (**C**). Data are presented as the mean ± standard error of the mean. A *p*-value < 0.05 was considered statistically significant. *p < 0.05, **p < 0.01.

The therapeutic effect of adiponectin was assessed using a mouse ITx model using 80 islet equivalents (IEQs). Before this assessment, preliminary investigations had demonstrated that ITx using 150 and 100 IEQs resulted in almost all the mice achieving normoglycemia (Supplemental Fig. 3A,B). A mild decrease in blood glucose levels was observed in both the adiponectin (+) and adiponectin (−) groups until POD 28. Thereafter, the decrease became more prominent, reaching a level that was nearly normal by POD 56 in the adiponectin (+) group (*p* = 0.026; Fig. 6A). The normoglycemia rate of the adiponectin (+) group was significantly higher than that of the adiponectin (−) group (66.7% vs. 18.2%, *p* = 0.023; Fig. 6B). Furthermore, blood glucose levels were elevated in all the mice in the adiponectin (+) group (*n* = 9) after graftectomy (210.11 ± 7.14 mg/dL vs. 767.56 ± 62.31 mg/dL, *p* < 0.0001; Fig. 6C). The therapeutic effect of adiponectin treatment was noticeable in the blood glucose and plasma insulin levels. The adiponectin (+) group maintained a higher level of plasma insulin than the adiponectin (−) group during the observation period, and there were significant differences at POD 14 (173.22 ± 30.92 pg/mL vs. 111.02 ± 15.05 pg/mL, *p* = 0.043) and POD 28 (197.79 ± 37.08 pg/mL vs. 121.84 ± 15.23 pg/mL, *p* = 0.035) (Fig. 6D). On the other hand, there was no significant difference in changes of blood glucose levels in the glucose tolerance test (GTT) at POD 56 between the adiponectin (+) and adiponectin (−) groups (*p* = 0.45; Supplemental Fig. 4A). Furthermore, no difference in the value of the area under the curve for blood glucose levels in the GTT (AUC-GTT) at POD 56 was noted between the two groups (768.75 ± 34.76 vs. 712.25 ± 8.49 mg/dL × h; Supplemental Fig. 4B).

Renal subcapsular ITx with an intraperitoneal injection of 1 mg/kg adiponectin was performed in two mice for further assessment. The blood glucose levels were not normalized during the observation period (Supplemental Fig. 5A). The AUC-GTT of this group was higher compared with the adiponectin (+) group (799.96 ± 22.96 vs. 1,037.63 ± 8.43 mg/dL × h, *p* = 0.0002; Supplemental Fig. 5B), indicating that a single injection of adiponectin did not improve ITx outcomes.

In summary, adiponectin treatment might reinforce the therapeutic effect of ITx, but its contribution appeared to be partial.

## Discussion

Adiponectin was one of the adipokines discovered in 1996^15^. Since then, its characteristics and roles have been investigated. Previous studies reported that adiponectin played a pivotal role in glucose and lipid metabolism^9,16^. For example, Yamauchi et al. revealed that adiponectin deficiency caused insulin resistance with glucose intolerance using genetically modified animals^9,17^. They also clarified that adiponectin activated 5′ AMP-activated protein kinase (AMPK) via AdipoR1 on skeletal muscle and improved glucose uptake, reduced gluconeogenesis, and increased insulin sensitivity in the tissue^18,19^. Furthermore, adiponectin directly affects pancreatic islets, exerting an antiapoptotic effect and improving the insulin-releasing function. Regarding the former, Jian et al. revealed that β-cell apoptosis induced by streptozotocin administration and a high-fat diet was attenuated in mice with adiponectin overexpression by preventing the cleavage of caspase 8, 9, and 3^12^. Regarding the latter, Okamoto et al. demonstrated that adiponectin administration induced an improvement in the insulin-releasing function of β cells in both in vitro and in vivo models^13^. The mechanism of these two effects of adiponectin relies on the activation of the phosphatidylinositol 3-kinase (PI3K)/protein kinase B (Akt) pathway and the mitogen-activated protein kinase/extracellular signal-regulated kinase (ERK) pathway via AdipoR1 and AdipoR2 rather than the AMPK pathway. Wijesekara et al. clarified some findings, including the expression of both AdipoR1 (prominently on skeletal muscle) and AdipoR2 (especially on the liver) on mouse pancreatic islets, the promotion of phosphorylation of both Akt and ERK in adiponectin-treated islets, and the prevention of insulin-releasing and antiapoptotic functions by Akt or ERK inactivation^20^. Similar findings were reported by Ye et al., and they also showed evidence that adiponectin contributed to islet regeneration via the expression of Nkx6.1 and MafA, which are relevant to differentiation to pancreatic β cells^21^.

These studies showed the possibility of adiponectin for ITx application with some advantages in the engraftment of transplanted islets and the regulation of blood glucose after engraftment. Du et al. previously demonstrated that adiponectin treatment protected cultured hypoxic islets from apoptosis, preserved insulin function, and improved ITx outcomes. They concluded that adiponectin treatment protected islets from ischemia–reperfusion injury by preventing the production of tumor necrosis factor-α^22,23^. However, the therapeutic effects of adiponectin in ITx have not been thoroughly investigated. The usefulness of visceral white adipose in the epididymal fat pad of mice in ITx was recently reported^14^. The ITx outcome was superior to that of intraperitoneal ITx (abdomen) and almost equal to renal subcapsular ITx in improving blood glucose and plasma insulin levels. Furthermore, neovascularization of the engrafted islets was most prominent in visceral white adipose tissue. However, vessel densities and the expression of angiogenic factors were the poorest compared with the other sites, indicating that white adipose tissue did not directly contribute to the angiogenesis of transplanted islets. Nevertheless, some factors derived from white adipose tissue might support engraftment. As a candidate supporting factor, we focused on adiponectin because it was confirmed that adiponectin-treated islets had higher expression levels of both angiogenic and adhesion factors^14^.

This study clarified some effects of adiponectin that may contribute to ITx reinforcement. First, adiponectin treatment improved insulin-releasing function, but this was dependent on glucose concentration. There was no difference between the adiponectin (+) and adiponectin (−) groups at low levels of glucose stimulation, but secretion was higher in the adiponectin (+) group at high levels of glucose stimulation. Furthermore, the insulin content in islets treated with adiponectin was high, and increased *Ins2* expression might support the effectiveness of adiponectin. These findings regarding the therapeutic effects of adiponectin on the insulin-releasing function of islets support the accuracy of previous studies^13,20^. The use of adiponectin is considered important because the improvement of the insulin-releasing function of islets (in other words, islets of high quality) may contribute to the success of ITx using a smaller number of islets, meaning that fewer donors will be required. Second, adiponectin treatment may promote islet engraftment via the induction of angiogenic and adhesion factors. We found that neovascularization of transplanted islets was prominent at POD 14 in the adiponectin (+) group. VEGF production in adiponectin-treated islets also increased. Furthermore, the expression of some angiogenic factor genes (*Vegf*s) was significantly higher in the adiponectin (+) group. Lee et al. also reported that adiponectin promoted *Vegfa* expression via adiponectin receptors and following PI3K/Akt pathway activation using a human chondrosarcoma cell line^24^. The importance of vascularization for the engraftment of transplanted islets has been well-discussed in many studies^25–27^. The expression of integrin β1 on islets was stimulated under adiponectin treatment at both the gene and protein levels. Integrin β1 is a receptor of various extracellular matrices, including collagen and laminin^28^. These extracellular matrices comprise the vascular endothelial basement membrane^29^, an engraftment site for transplanted islets in cases of intraportal ITx. Thus, integrin β1 may contribute to the engraftment of transplanted islets by promoting adhesion to the transplant site.

Regarding transplantation data, adiponectin treatment improved the therapeutic effect of ITx, and this was reflected in the blood glucose level. The long-lasting improvement in the blood glucose level was confirmed in the adiponectin (+) group. We speculate that the acute adiponectin treatment promoted islet engraftment by angiogenesis and adhesion to the transplant site by the expression of angiogenic and adhesion factors. This resulted in the successful engraftment of more islets in the adiponectin (+) group compared with the adiponectin (−) group. In turn, this might lead to the better outcome of a long-lasting improvement in blood glucose levels. On the other hand, there was no significant difference between adiponectin (+) and adiponectin (−) groups in the GTTs at POD 56. We suggest that this discrepancy is due to the method of adiponectin administration. This study administered adiponectin to isolated islets temporally by overnight culture. The engraftment of the islets may be promoted via the angiogenic and adhesion effects of adiponectin, and as a result, long-lasting blood glucose improvement was achieved. However, the temporal adiponectin treatment was insufficient to achieve a complete cure of the diabetic condition, and this was reflected in the GTT results. In other words, temporal treatment of adiponectin improves the therapeutic effect of ITx in ameliorating blood glucose, but this method is insufficient to show the usefulness in long-term follow-up GTTs. Continuous administration of adiponectin may strengthen the therapeutic effect of ITx and cure the diabetic condition. Advancements in the methodology, including a gene-editing system and the development of a method for the mass production of new recombinant adiponectin at a lower price, will elucidate more details of the therapeutic effects of adiponectin for ITx so that this treatment can be applied to clinical settings. For example, adiponectin can be one of the supplements included in the solution for islet isolation and transplantation. The isolation process is a harsh event for islets because they suffer from ischemia, and digestion by pancreatic enzymes also injures them in this process. Thus, adiponectin treatment can prevent the loss of islets in the isolation process and promote transplantation engraftment.

In conclusion, adiponectin treatment had preferable effects in the insulin-releasing, angiogenic, and adhesion functions of islets. Adiponectin contributed to the improvement of ITx outcomes. The method of adiponectin treatment should be investigated for use in future clinical settings.

## Methods

### Animals and ethical statement

We used 8- to 12-week-old male C57BL/6J mice (CLEA Japan Inc., Tokyo, Japan) as syngeneic islet donors and diabetic recipients. The mice were housed under specific pathogen-free conditions and had access to food and water ad libitum. There was no fasting treatment before a challenge or assessment except before GTTs. The care of mice and experimental procedures complied with the principles of laboratory animal care (Guide for the Care and Use of Laboratory Animals, National Institutes of Health publication 86-23, 1985). The experimental protocol was approved by the Animal Care and Use Committee of Fukuoka University (approval number: 2010058).

### Islet isolation

The islets were isolated using a collagenase digestion and purification protocol modified from the original method of Gotoh et al.^30^ Briefly, pancreatic tissue was digested at 37 °C for 18 min using 1 mg/mL collagenase V (Sigma-Aldrich, St. Louis, MO, USA) solution. Then, the islets were isolated using Biocoll Separating Solution (Biochrom GmbH, Berlin, Germany) at density gradients of 1.100, 1.083, 1.077, and 1.040 and centrifugation at 840×*g* for 13 min. Approximately 100% purity was guaranteed using a 70 µm Cell Strainer (Corning Inc., Corning, NY, USA) and handpicking under a microscope.

### Adiponectin treatment

Isolated islets were cultured overnight in low glucose Dulbecco’s modified Eagle medium (DMEM; Gibco, Tokyo, Japan) with 0.2% bovine serum albumin (Sigma-Aldrich) and 1% penicillin–streptomycin solution (Invitrogen, Waltham, MA, USA). Fetal bovine serum was not used because it contains some growth factors that may ameliorate the condition of isolated islets. The islets were treated with the addition of 10 µg/mL recombinant mouse adiponectin (Adiponectin Mouse HEK293; BioVendor Research and Diagnostic Products, Brno, Czech Republic) to the culture medium (adiponectin (+) group). The dose of adiponectin was determined according to previous publications^22,30^. Islets without adiponectin treatment (adiponectin (−) group) were prepared as the control group.

### Real-time reverse-transcription polymerase chain reaction analysis (qRT-PCR)

Total RNA was extracted from > 500 islets using TRIzol Reagent (Invitrogen) and a PureLink RNA Mini Kit (Invitrogen) as per the manufacturer’s instructions. Reverse transcription was performed using a QuantiTect Reverse Transcription Kit (QIAGEN K.K., Tokyo, Japan). qRT-PCR analysis was carried out on a CFX Connect Real-Time PCR Detection System (Bio-Rad Laboratories, Inc., Hercules, CA, USA) with THUNDERBIRD SYBR qPCR Mix (Toyobo Co., Ltd., Osaka, Japan). All primers were designed by Fasmac Corp., Ltd. (Atsugi, Japan) (Table 1). The results were normalized to the β-actin housekeeping gene. Data were presented as the fold difference over the detectable cycle threshold (Ct) value, which was calculated using the ΔΔCt method.

**Table 1:** Primers used for qRT-PCR analysis.

Primer	Sequence (5′–3′)	Tm (°C)
*Actb* F	CATCCGTAAAGACCTCTATGCCAAC	67.2
*Actb* R	ATGGAGCCACCGATCCACA	69.0
*AdipoR1* F	CCGTCCGGGCAGTACACT	61.4
*AdipoR1* R	ATCTGTGAAGGAGCAGCAG	57.1
*AdipoR2* F	CACCGGAGCTGCCCTCTA	60.8
*AdipoR2* R	AGTGAAACCAGATGTCACA	53.9
*Ins2* F	TCAAGCAGCACCTTTGTGGTT	63.1
*Ins2* R	TCCACCCAGCTCCAGTTGT	61.7
*Itgb1* F	CCATGCCAGGGACTGACAGA	64.7
*Itgb1* R	GAGCTTGATTCCAATGGTCCAGA	64.8
*Itgb2* F	GCATCTGTGGGCAGTGTGTA	60.8
*Itgb2* R	ATTTGCCACAGTTGCAGGA	60.3
*Vegfa* F	ACATTGGCTCACTTCCAGAAACAC	67.2
*Vegfa* R	TGGTTGGAACCGGCATCTTTA	68.2
*Vegfb* F	CACTGGGCAACACCAAGTC	64.4
*Vegfb* R	TGTCTGGCTTCACAGCACTC	64.4
*Vegfc* F	CAGTGCATGAACACCAGCACA	68.5
*Vegfc* R	TAGACATGCACCGGCAGGAA	68.9

*Tm* melting temperature, *F* forward, *R* reverse.

### GSIS and measurement of insulin content in islets

GSIS was assessed using 10 IEQs (150 µm-sized islets). They were preincubated with 3.3 mM glucose for 60 min and then stimulated with glucose at concentrations of 3.3 mM (low glucose) and 16.5 mM (high glucose) for 60 min. The amount of insulin in the culture supernatants was measured by enzyme-linked immunosorbent assay (ELISA) using a Mouse Insulin ELISA Kit (RTU) (FUJIFILM Wako Shibayagi Corp., Shibukawa, Japan). The plates were read using an iMark Microplate Absorbance Reader (Bio-Rad Laboratories, Inc.) with Microplate Manager v6.3 software (Bio-Rad Laboratories, Inc.). Then, the stimulation index, defined as the ratio of released insulin at conditions of high versus low glucose stimulation, was calculated. The ELISA kit was also used to measure the insulin content of the islets by extracting insulin from 10 IEQs using 1 mL radioimmunoprecipitation assay (RIPA) buffer (FUJIFILM Wako Pure Chemical Corporation, Osaka, Japan) containing 100× protease inhibitor cocktail (Nacalai Tesque, Kyoto, Japan) and a phosphatase inhibitor cocktail (Nacalai Tesque).

### Cellular viability of islets

The islets were stained with Hoechst 33342 and propidium iodide (PI) (Thermo Fisher Scientific, Waltham, MA, USA). The cellular viability of the islets was calculated as follows: (Hoechst 33342-positive cells − PI-positive cells) / Hoechst 33342-positive cells) × 100 (%).

### Quantification of internal VEGF and integrin β1 in islets

The quantification of VEGF, an angiogenic factor, and integrin β1, a receptor for various extracellular matrices including collagen, fibronectin, and laminin, in islets was performed using an ELISA kit. Briefly, the islets were cultured for 24 h in low glucose DMEM with 0.2% bovine serum albumin and 1% penicillin–streptomycin solution, with or without adiponectin, at 37 °C with 5% CO_2_. For VEGF quantification, 50 islets were dissolved in 100 µL RIPA buffer with protease and phosphatase inhibitor cocktails. The lysates were collected as samples for use with a Mouse VEGF Detection Assay Kit (Chondrex, Inc., Woodinville, WA, USA) as per the manufacturer’s instructions. For integrin β1 quantification, 10 islets were seeded on a Costar 96-well cell culture plate (Corning, Glendale, AZ, USA) treated with poly-l-lysine (Sigma-Aldrich) and cultured for 24 h in low glucose DMEM with 0.2% bovine serum albumin and 1% penicillin–streptomycin solution, with or without adiponectin, at 37 °C in 5% CO_2_. CytoGlow Integrin β1 (Assay Biotechnology, Fremont, CA, USA) was used for integrin β1 quantification following the manufacturer’s instructions. A primary antibody (rabbit polyclonal anti-integrin β1 antibody or mouse monoclonal anti-GAPDH antibody) was used as an internal control, and the samples were incubated at 4 °C for 16 h after treatments to inactivate endogenous peroxidase and block nonspecific binding. Following the addition of horseradish peroxidase-conjugated secondary antibody, TMB substrate was added. The resultant color reaction was measured by determining the OD_450_ using an iMark Microplate Absorbance Reader with Microplate Manager v6.3 software.

### Diabetes induction in recipient mice

Diabetes was induced in recipient mice by a single intravenous injection of streptozotocin (180 mg/kg body weight; Sigma-Aldrich). The blood glucose levels of the mice were measured using a Glutest Mint glucose analyzer (Sanwa Kagaku Kenkyusho Co. Ltd., Nagoya, Japan) at 5–7 days after the injection. Mice with blood glucose levels > 400 mg/dL were used as diabetic recipients.

### ITx and monitoring

We transplanted 80 IEQs into the renal subcapsular space of the left kidney in diabetic mice under general anesthesia using isoflurane (FUJIFILM Wako Pure Chemical Corporation). The engraftment or rejection of the transplanted islets was assessed by monitoring blood glucose and plasma insulin levels and changes in blood glucose levels in GTTs. Nonfasting blood glucose levels were measured from 8 to 10 am on POD 0 (i.e., pretransplantation), 1, 3, 5, 7, 10, 14, 17, 21, 24, 28, 35, 42, 49, and 56. Normoglycemia was defined as a blood glucose level < 200 mg/dL. Blood samples (200 µL) for plasma insulin were collected from the tail veins of the mice on PODs 0, 3, 7, 14, 28, and 56. The plasma was separated from the blood (approximately 80 µL plasma was obtained from each blood sample) and preserved at − 80 °C until insulin measurement using a Mouse Insulin ELISA Kit (RTU). Intraperitoneal GTTs (IPGTTs) were performed to the mice achieved normoglycemia by ITx 2 months after transplantation by injecting glucose solution (2 g/kg body weight) into the peritoneal cavity after 10–12 h of fasting. Then, the glucose levels were measured at 0, 30, 60, 90, and 120 min after the injection. The AUC-GTTs were also calculated.

### Graftectomy

After assessing the therapeutic effect of the ITx, the left kidneys were recovered from the mice in both the adiponectin (+) and adiponectin (−) groups under general anesthesia. The left nephrectomy procedure involved ligation of the left renal artery and vein at the renal hilum. Blood glucose levels in the adiponectin (+) group were measured before and after the graftectomy (at 7 days after graftecotmy).

### Histologic assessment

Kidney and samples of isolated islets were histologically assessed. The isolated islets were embedded in 2% UltraPure LMP Agarose (Invitrogen) gel. The organs and tissues were fixed in a 10% neutral formalin buffer solution and embedded in paraffin. Paraffin sections (3 μm) of kidney specimens were either stained with hematoxylin and eosin or underwent immunohistochemistry staining to determine the presence of insulin (to detect islets) and vWF (to detect vessels). The primary antibodies were guinea pig anti-insulin (1:100; Agilent Technologies, Santa Clara, CA, USA), rabbit anti-vWF antibody (1:100; Abcam, Cambridge, UK), rabbit anti-AdipoR1 (1:1000; Abcam), and goat anti-AdipoR2 (1:250; Abcam). After incubation with the primary antibodies, Alexa 488-conjugated donkey anti-guinea pig, Alexa 647-conjugated donkey anti-rabbit, Alexa 488-conjugated goat anti-rabbit, and Cy3-conjugated goat anti-rabbit (all 1:100; Jackson ImmunoResearch Laboratories, Inc., West Grove, PA, USA) as well as Alexa 488 anti-goat and Cy3 anti-goat were used as secondary antibodies. Nuclear staining was performed using 4′,6-diamidino-2-phenylindole (DAPI). All histology samples were observed under a BZ-X700 microscope (Keyence, Itasca, IL, USA), and the findings were quantified for statistical evaluation. The vessel density in the engrafted islets was calculated using the following formulas: (numbers of vessels [vWF-positive] in the islet/area of the islet in mm^2^) and (vWF-positive area/area of the islet) in a field of view at × 200 magnification using ImageJ software (National Institutes of Health, Bethesda, MD, USA). AdipoR1- or AdipoR2-positive areas per islet (percentage islets expressing AdipoR1 and AdipoR2, respectively) were quantified using the same method.

### Statistical analysis

Blood glucose, plasma insulin, and changes in blood glucose in the IPGTTs were compared between adiponectin (+) and adiponectin (−) groups using two-way repeated-measures analysis of variance. Comparisons between the two groups for other parameters were performed using the Student *t*-test. The Kaplan–Meier method with the log-rank test was used to compare the rate of normoglycemia. All data were presented as means ± standard error of the mean. Significant differences were defined as *p* < 0.05. All tests were two-sided, and all statistical analyses were performed using JMP v12.0.0 software (SAS Institute Inc., Cary, NC, USA).

### Statement on ARRIVE guidelines

This study was reported in accordance with the ARRIVE guidelines. All experiments were performed in accordance with relevant guidelines and regulations.

## Supplementary Information

Supplementary Legends.

Supplementary Figures.
