# Supplementary material for: Possibility of adiponectin use to improve islet transplantation outcomes
Source: Sci Rep. 2022 Jan 10;12:444. doi: 10.1038/s41598-021-04245-0 (PMC8748684; doi:10.1038/s41598-021-04245-0)
Supplement: Supplementary file 1 — Supplementary Legends. [file 41598_2021_4245_MOESM1_ESM.docx]

**Figure legends**

**Supplemental Figure 1. AdipoR2 expression on isolated islets. A** *AdipoR2*expression in islets with adiponectin treatment (adiponectin (+), blue) and without adiponectin treatment (adiponectin (−), (orange) was quantified by qRT-PCR. The results were normalized to the ꞵ-actin housekeeping gene and were presented as the fold difference over the detectable Ct value, which was calculated using the ΔΔCt method. **B** Immunohistochemical findings of isolated islets in adiponectin (+) group (left) and adiponectin (−) (right) stained for AdipoR2 (red) and insulin (green), nuclear (blue, using DAPI). Scale bar: 100 µm. **C** Quantification of AdipoR2-positive area per islet (percentage islets expressing AdipoR2). Data are presented as the means ± standard error of the mean. A *p*-value <0.05 was considered statistically significant.

**Supplemental Figure 2. Angiogenesis in transplanted islets. A** Immunohistochemical findings of transplanted islets in the renal subcapsular space at POD 56. Islets treated with adiponectin (adiponectin (+), left) and without adiponectin (adiponectin (−), right) were stained for insulin (green) and vWF (red). DAPI was used for nuclear staining (blue). **B** Vessel densities, defined as the number of vWF-positive capillaries per islet area, at POD 56. **C** Vessel densities, defined as the vWF-positive area per islet area, at POD 56. Data are presented as the means ± standard error of the mean. A *p*-value < 0.05 was considered statistically significant.

**Supplemental Figure 3. Blood glucose level after 150 and 100 IEQs ITx. A** Individual blood glucose level after 150 IEQs ITx. **B** Individual blood glucose level after 100 IEQs ITx. Blue: adiponectin (+) group; Orange: adiponectin (–) group.

**Supplemental Figure 4. The therapeutic effect of treatment of adiponectin in ITx in GTT.** Assessment of data from the GTT at 2 months after transplantation in adiponectin (+) (*n* = 6) and adiponectin (−) (*n* = 2) using change of blood glucose level (**A**) and area under the curve calculated by multiplication of blood glucose and time (AUC-GTT) (**B**). GTT was performed to the mice achieved normoglycemia by ITx. Data are presented as the means ± standard error of the mean. A *p*-value <0.05 was considered statistically significant.

**Supplemental Figure 5. The therapeutic effect by intraperitoneal injection of adiponectin in ITx. A** Changes of blood glucose level of two mice received islet transplantation with intraperitoneal injection of adiponectin. **B** AUC-GTT of the mice compared with adiponectin (+) group. Data are presented as the means ± standard error of the mean. A *p*-value <0.05 was considered statistically significant.
