# Supplementary material for: Possibility of adiponectin use to improve islet transplantation outcomes
Source: Sci Rep. 2022 Jan 10;12:444. doi: 10.1038/s41598-021-04245-0 (PMC8748684; doi:10.1038/s41598-021-04245-0)

Supplemental Figure 1

A

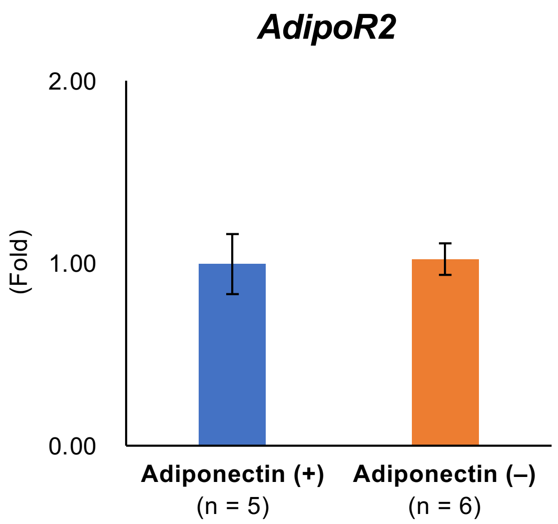

B

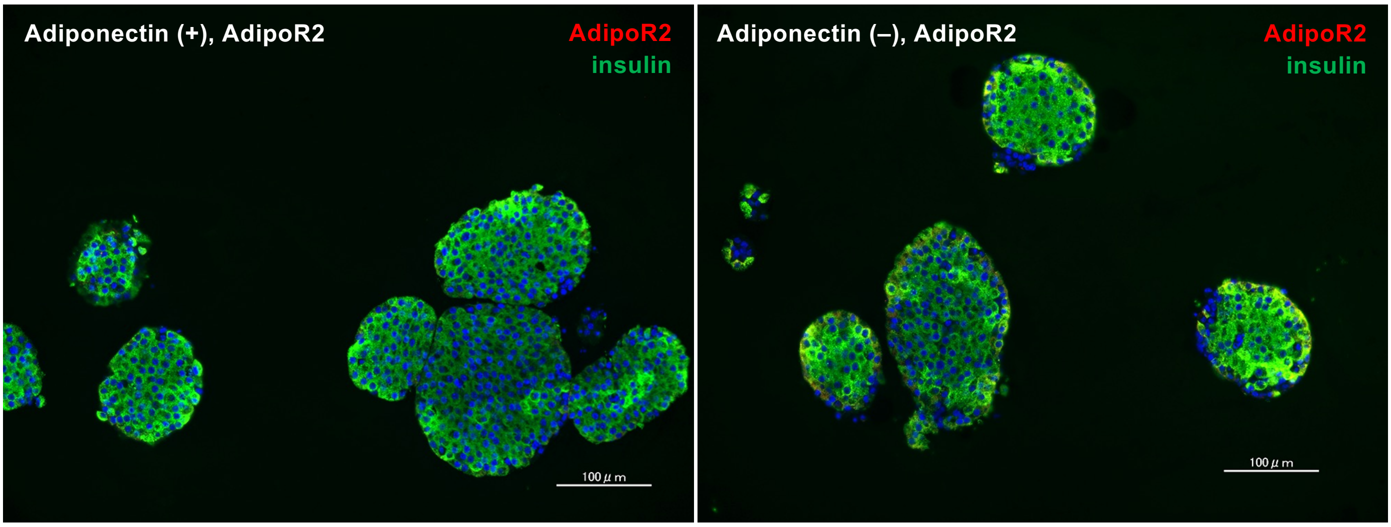

C

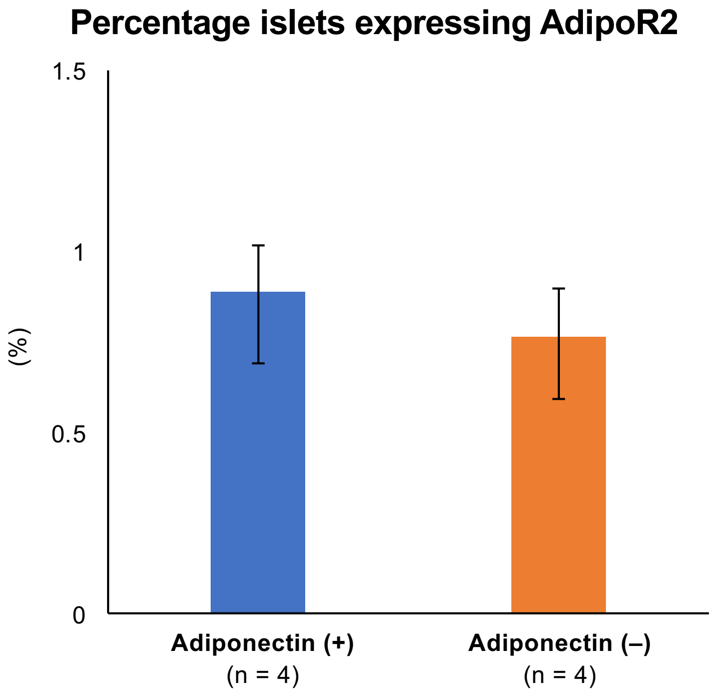

Supplemental Figure 2

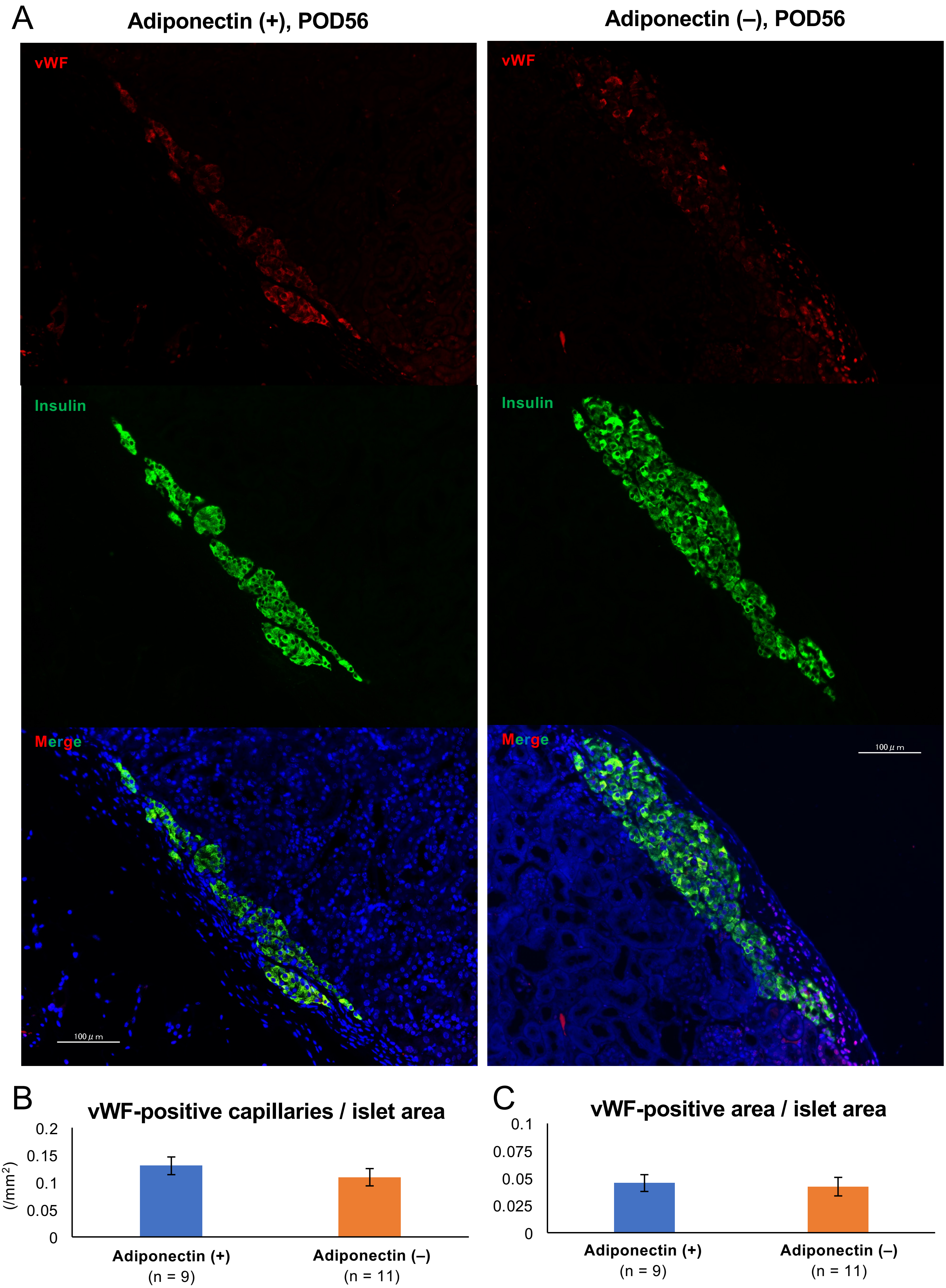

Supplemental Figure 3

A

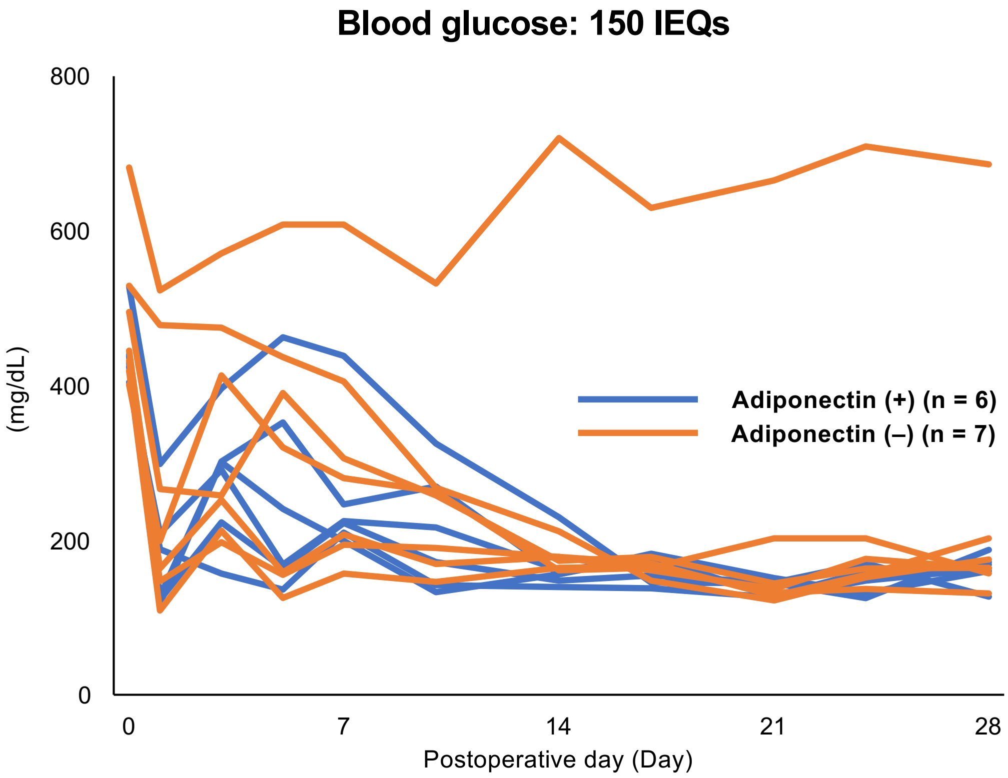

B

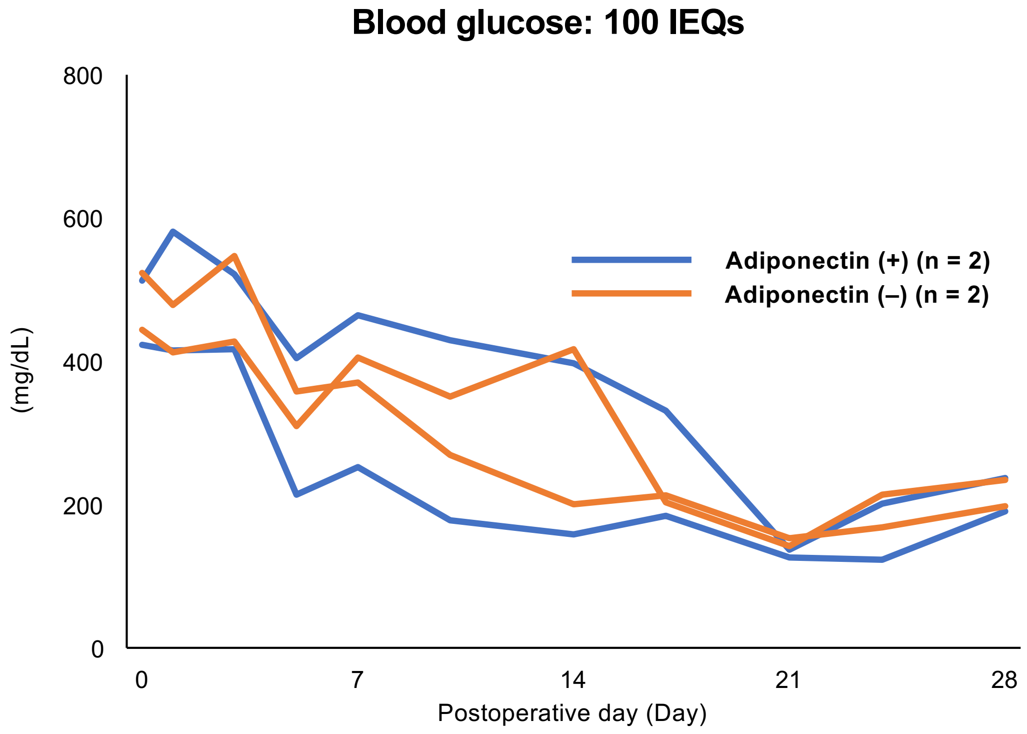

Supplemental Figure 4

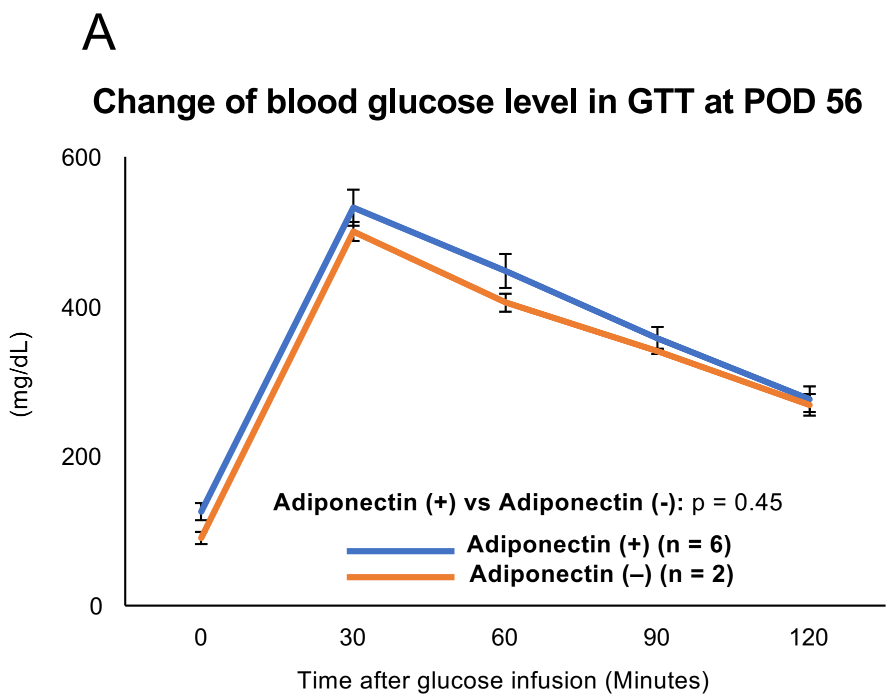

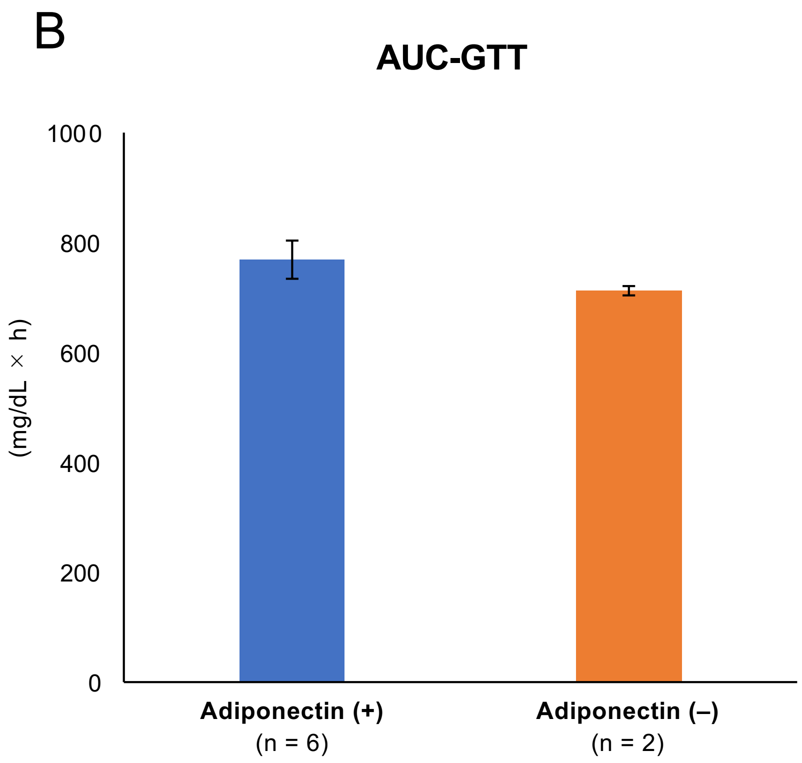

Supplemental Figure 5

A

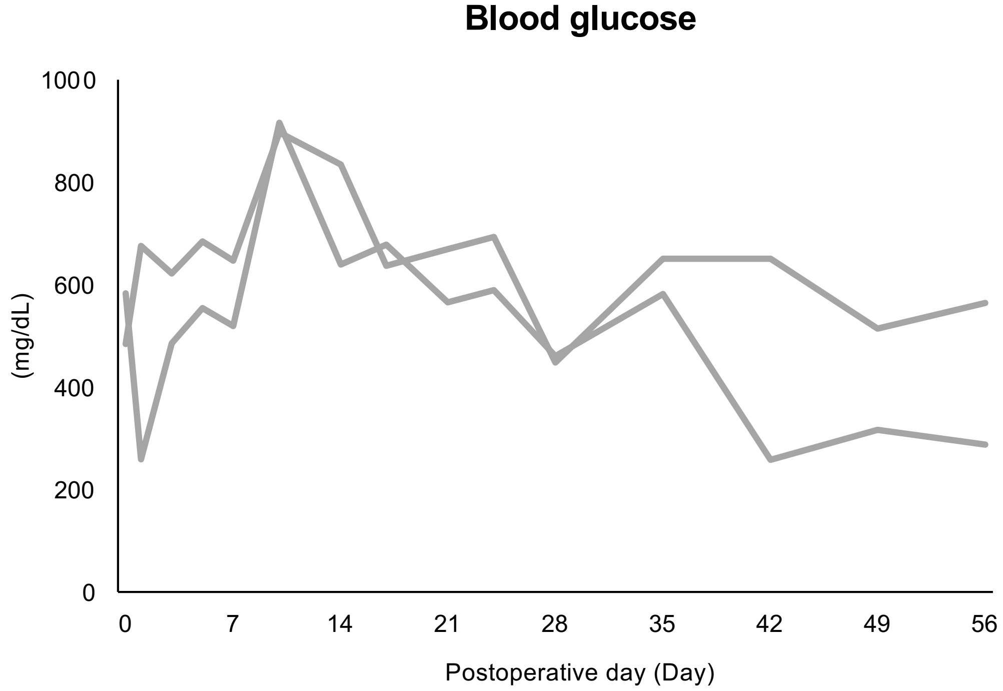

B

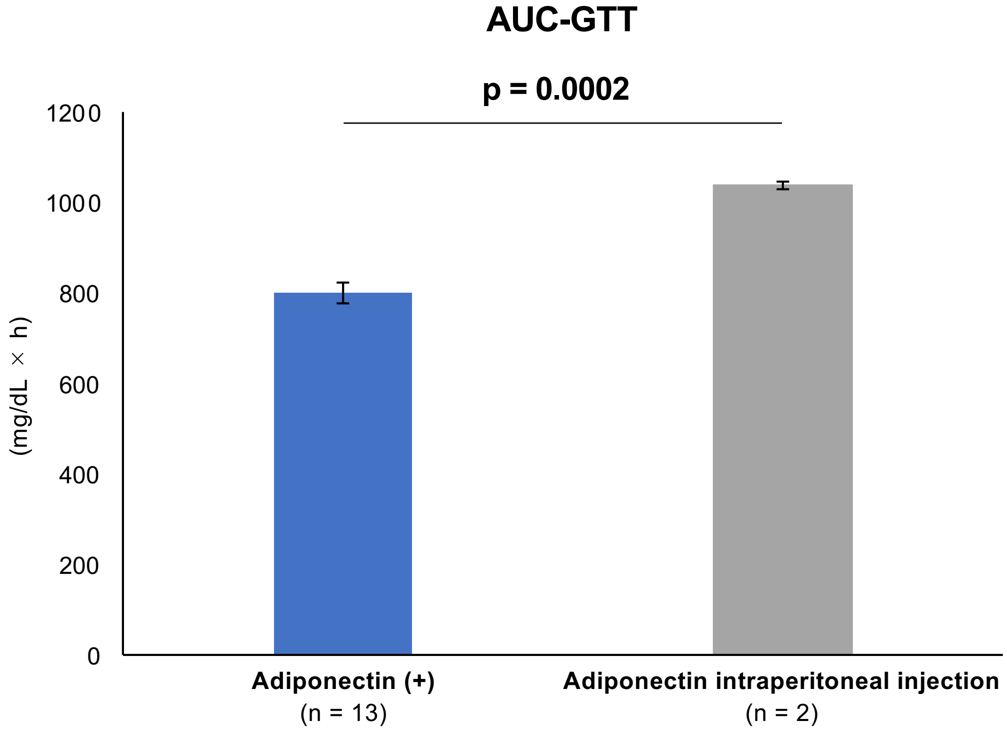

Supplement: Supplementary file 2 — Supplementary Figures. [file 41598_2021_4245_MOESM2_ESM.pdf]
